# Underwater acoustic metamaterials

**DOI:** 10.1093/nsr/nwac246

**Published:** 2022-11-03

**Authors:** Erqian Dong, Peizheng Cao, Jinhu Zhang, Sai Zhang, Nicholas X Fang, Yu Zhang

**Affiliations:** Key Laboratory of Underwater Acoustic Communication and Marine Information Technology of the Ministry of Education, College of Ocean and Earth Sciences, Xiamen University, Xiamen 361005, China; State Key Laboratory of Marine Environmental Science, Xiamen University, Xiamen 361005, China; Key Laboratory of Underwater Acoustic Communication and Marine Information Technology of the Ministry of Education, College of Ocean and Earth Sciences, Xiamen University, Xiamen 361005, China; State Key Laboratory of Marine Environmental Science, Xiamen University, Xiamen 361005, China; Key Laboratory of Underwater Acoustic Communication and Marine Information Technology of the Ministry of Education, College of Ocean and Earth Sciences, Xiamen University, Xiamen 361005, China; State Key Laboratory of Marine Environmental Science, Xiamen University, Xiamen 361005, China; School of Physics and Electronic Engineering, Jiangsu University, Zhenjiang 212013, China; Department of Mechanical Engineering, Massachusetts Institute of Technology, Cambridge, MA 02139, USA; Key Laboratory of Underwater Acoustic Communication and Marine Information Technology of the Ministry of Education, College of Ocean and Earth Sciences, Xiamen University, Xiamen 361005, China; State Key Laboratory of Marine Environmental Science, Xiamen University, Xiamen 361005, China

**Keywords:** underwater acoustic metamaterials, invisibility cloaking, beam formation, topological acoustics, metasurfaces, absorbers

## Abstract

Acoustic metamaterials have been widely investigated over the past few decades and have realized acoustic parameters that are not achievable using conventional materials. After demonstrating that locally resonant acoustic metamaterials are capable of acting as subwavelength unit cells, researchers have evaluated the possibility of breaking the classical limitations of the material mass density and bulk modulus. Combined with theoretical analysis, additive manufacturing and engineering applications, acoustic metamaterials have demonstrated extraordinary capabilities, including negative refraction, cloaking, beam formation and super-resolution imaging. Owing to the complexity of impedance boundaries and mode transitions, there are still challenges in freely manipulating acoustic propagation in an underwater environment. This review summarizes the developments in underwater acoustic metamaterials over the past 20 years, which include underwater acoustic invisibility cloaking, underwater beam formation, underwater metasurfaces and phase engineering, underwater topological acoustics and underwater acoustic metamaterial absorbers. With the evolution of underwater metamaterials and the timeline of scientific advances, underwater acoustic metamaterials have demonstrated exciting applications in underwater resource development, target recognition, imaging, noise reduction, navigation and communication.

## Teaser

This review summarizes the developments in underwater acoustic metamaterials over the past 20 years and discusses the prospect in cloaking, beam formation, hologram, communications, sound absorption, ocean detection, and sensing.

## INTRODUCTION

Oceans cover nearly three-quarters of the Earth’s surface and contain 97% of the planet’s total water resources. It is well known that light beams are significantly attenuated by the scattering of numerous particles in the sea, which makes light and radar-based communication and navigation virtually useless, except for very short distances in the ocean. Sound propagates more efficiently in rivers and oceans, and thus may deliver information over a larger spatial scale than most sensory cues such as chemicals and electromagnetic waves. Given the rapid development of underwater intelligent sensing technology and ocean development, there is an urgent need to understand the current development of underwater functional materials for sound control.

In the past few decades, underwater acoustic metamaterials have drawn considerable attention in underwater acoustic wave manipulation, including underwater acoustic beam control, asymmetric transmission, subwavelength imaging and water–air interface coupling. In 2000, researchers proposed a locally resonant material that acted as a subwavelength building block structure with negative elastic constants [1]. The structured unit cells’ local resonant property induces the band structure, which breaks the classical mass density law by tuning the effective stiffness and mass density of the metamaterials. It was also pointed out that the realization of low-frequency sound insulation could be equivalent to using the negative mass density near the transmission valley frequency. The principle of the acoustic negative mass density based on local resonance paves the new way for other types of acoustic metamaterials.

In 2006, an underwater acoustic metamaterial with the negative acoustic bulk modulus was created based on Helmholtz resonators, and the aforementioned negative modulus was theoretically and experimentally observed [2]. Generally, double negative acoustic metamaterials are typically categorized as metamaterials with a negative mass density and acoustic bulk modulus. Double-negative metamaterials have been established by combining two types of single-negative materials at the same frequency. In 2007, double-negative metamaterials were theoretically proposed using a double-unit structure [3]. One special aspect of the double-negative metamaterial is the strong monopolar resonance, whereas the negative bulk modulus occurs from the water spheres that include cavities. The other special aspect is the strong dipolar resonance, in which the rubber-coated composite sphere in the epoxy creates a negative mass density. Since the first attempt to utilize subwavelength building blocks for unusual acoustic properties, an increasing number of acoustic metamaterials have been developed.

The effective medium theory in fluid or air can be employed to describe the dynamic parameters of artificial composite structures [4–11]. Initially, acoustic metamaterials were divided into several types, including locally resonant metamaterials [1,12], membrane-type metamaterials [13–15], coiling-space metamaterials [12,16–19] and gradient-index metamaterials [20,21]. In recent decades, black hole metamaterials [22–26], topological metamaterials [27–29], non-Hermitian metamaterials [30–32] and graphene-like metamaterials [33–35] have attracted considerable attention. Furthermore, acoustic metamaterials are not restricted to local resonant components as well as periodic structures; materials with unit cells that exhibit homogeneous acoustic properties under long-wave approximation conditions are classified as acoustic metamaterials.

However, in underwater acoustic applications, acoustic metamaterials are not well understood because of the following challenges. (1) The acoustic impedance of water is approximately 3600 times that of air. Therefore, common metals such as steel, aluminum, lead, epoxy resin and photosensitive resin produced by three-dimensional (3D) printing can no longer be used as rigid boundaries, and both longitudinal and transverse waves should be considered in the elastic modulus. (2) Density in water and acoustic-solid coupling cannot be ignored, and the mode evolution between solid structures and water should be considered in the design of underwater metamaterials. When considering the dynamic density of a composite structure with a fluid matrix and inclusions, the effective dynamic mass density of composites has been proven analytically and numerically under long-wave approximations [7]. The effective parameters of elastic columns contained in non-viscous liquid and air have also been thoroughly investigated [36,37].

**Figure 1.**
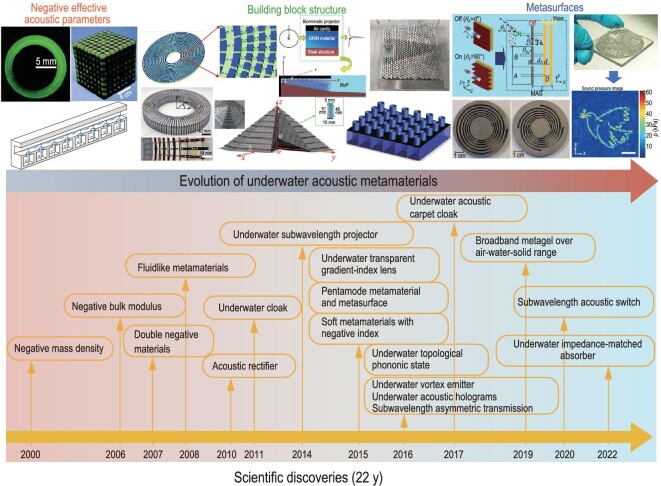
The evolution of underwater metamaterials and timeline of scientific advances. Firstly, researchers focused on the negative effective acoustic parameters of the metamaterials. They showed effective negative bulk modulus [2], effective negative mass density [1], and double negative parameters [3] that lay the foundation for achieving versatile functionalities. The subwavelength building block structures can achieve acoustic invisibility and beam control. Cloaking is a most representative example of hiding objects or opening a virtual hole in a wall, including invisibility cloaking [42,43] and carpet cloaking [44]. To achieve underwater beam shift and target detection, underwater conformal transformation acoustics [45] have been proposed. On the basis of the transformation acoustics, a kind of complementary metamaterial [46] has been proposed for penetrating aberration layers in biomedical imaging applications. In comparison with traditional tunable phased arrays, acoustic metasurfaces can be known as passive subwavelength unit cells with wavefront-shaping capabilities in phase engineering. Underwater acoustic rectifiers [47] and subwavelength acoustic switches [48] have been introduced to realize the on-and-off propagation of underwater asymmetric transmission [49]. An underwater acoustic vortex generator using multi-arm coiling slits is capable of producing acoustic vortex beams through regulating the transmission phase and amplitude [50]. Recent proposed acoustic holograms as underwater acoustic metasurfaces in phase engineering can achieve a hundred times more reconstruction freedom than conventional phased-array devices [51]. From left to right: photographs were adapted from [1,2,44–46,48,50,51].

In this study, we evaluated the developments in underwater metamaterials, including underwater acoustic invisibility cloaking, beam formation, metasurfaces, phase engineering, topological acoustics, and metamaterial absorbers. The evolution of underwater metamaterials and the timelines of novel and unique scientific discoveries over the past two decades are shown in Fig. 1.

## UNDERWATER INVISIBILITY CLOAKING

### Underwater transformation acoustics

Pendry *et al.* [38] and Leonhardt [39] initially conceived the theory of cloaking of electromagnetic waves that are built on the form equivalence of Maxwell’s equations, which was subsequently confirmed using experimental results at the microwave frequency band [40]. Coordinate transformation was further demonstrated as an instructive tool for freely controlling the light beam and electromagnetic waves, which has drawn a lot of attention for designing intriguing devices. Using the form equivalence of Maxwell’s equations, the concept of transformation optics can be expanded to include elastodynamics and acoustics. A universal elastodynamic equation of motion was proposed by Milton *et al.*, which was immutable under intricate transformation elastodynamics [41]. In 3D geometric structures, the equations for DC conductivity and the acoustic field were compared. By determining the immutability of the acoustic equation, the bulk modulus and density necessary for attaining cloaking were derivable [42]. The time-harmonic acoustic equation is expressed as


(1) \begin{eqnarray*} \nabla \cdot [\mathop \rho ^ \leftrightarrow {(x)^{ - 1}}\nabla p(x)]=-[{\omega ^2}/\kappa (x)]p(x), \end{eqnarray*}

where $\mathop \rho \limits ^ \leftrightarrow (x)$ denotes the mass density tensor, κ(*x*) represents the bulk modulus and *p*(*x*) signifies the pressure field. Through mapping the coordinate position x in physical space to the corresponding coordinate position *x*′(*x*) in virtual space, in which *p*′[*x*′(*x*)] = *p*(*x*), the acoustic equation becomes


(2) \begin{eqnarray*} {\nabla ^{\prime }}\bigg [\frac{1}{{{\rho ^{\prime }}({x^{\prime }})}}{\nabla ^{\prime }}{p^{\prime }}({x^{\prime }})\bigg ] = - \frac{{{\omega ^2}}}{{{\kappa ^{\prime }}({x^{\prime }})}}{p^{\prime }}({x^{\prime }}). \end{eqnarray*}

By introducing the Jacobian transformation matrix with elements, ${A_{ki}} = {{\partial x_k^{\rm {{\prime }}}}}/{{\partial {x_i}}}$, the new transformation equation shows that the mass density ${\mathop {\rho ^{\prime }}\limits ^\leftrightarrow (x^{\prime })}$ and bulk modulus *k*′(*x*) in the transformed space are related to the original space $\mathop \rho \limits ^ \leftrightarrow (x)$ and *k*(*x*) based on the Jacobian transformation matrix, which is


(3) \begin{eqnarray*} 1/[{\rho ^{\prime }}({x^{\prime }})] = A[1/[\rho (x)]]{A^T}/\det A \end{eqnarray*}

and


(4) \begin{eqnarray*} {\kappa ^{\prime }}({x^{\prime }}) = \kappa (x)\det A. \end{eqnarray*}

These equivalence relations are described as acoustic wave propagations in complex materials with acoustic bulk modulus *k*′(*x*) and mass density $\mathop {\rho ^{\prime }}\limits ^\leftrightarrow (x^{\prime })$ that can guide the acoustic wave as if they are in a curved space. The 3D acoustic cloaking system employs linear radial transformation *r*′ = *a* + *r*(*b* − *a*)/*b*. The relative mass density elements and bulk modulus are


\begin{eqnarray*} \rho _r^{\prime } &=& \frac{{b - a}}{b}\frac{{{r^{^{\prime }2}}}}{{{{({r^{\prime }} - a)}^2}}}, \quad \rho _\theta ^{\prime } = \rho _\varphi ^{\prime } = \frac{{b - a}}{b}, \\ && \text{and}\quad {\kappa ^{\prime }} = \frac{{{{(b - a)}^3}}}{{{b^3}}}\frac{{{r^{^{\prime }2}}}}{{{{({r^{\prime }} - a)}^2}}}. \end{eqnarray*}

However, the 3D acoustic cloaking requires an anisotropic mass density and infinite parameters, which are uncommon in naturally occurring materials. As a result, the difficulty of adequate material fabrication has hindered experimental research concerning acoustic cloaking, which remains challenging to date. The realization of acoustic cloaking relies on acoustic metamaterials that have been designed using the local resonance method [52–55]. On the basis of effective medium theory, multi-layered structured acoustic cloaking with homogeneous metamaterials was also employed [6,56].

**Figure 2.**
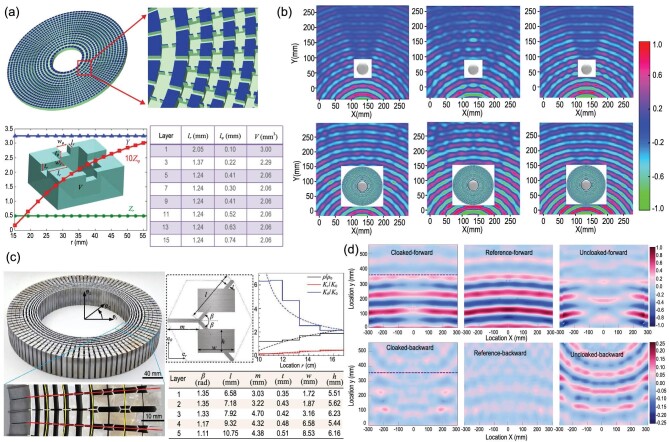
(a) Configuration (shunt capacitors and serial inductors) of cylindrical acoustic cloaking, which is synthesized by a sound transmission line. Every unit cell for the acoustic circuit’s one building block comprises one large central cavity with channels connecting to four adjacent blocks. The design adopts reduced cloaking parameters. (b) Pressure field measurement mappings for the cloaked and bare steel cylinders illuminated using a point source of ultrasound. Located in the water tank center, the cloaking encompasses the steel cylinder. In the case of the bare steel cylinder, the scattering field patterns are separate at 60, 52 and 64 kHz. (c) The detailed photograph and material parameters of the designed solid acoustic cloak by pentamode metamaterials. (d) The measured sound pressure fields include the backscattering and forward transmitted pressure for cloaked, uncloaked and reference cases. Panels (a) and (b) were adapted based on permission from Zhang *et al.*, Phys Rev Lett 106, 024301 (2011). Copyright 2011, American Physical Society. Panels (c) and (d) were adapted based on permission from Chen *et al.*, Phys Rev B 95, 180104 (2017). Copyright 2017, American Physical Society.

The first practical broadband acoustic cloaking for underwater ultrasound was later proposed and constructed using the network of acoustic circuit elements [43]. Moreover, 2D acoustic cloaking can lower the visibility of hidden objects from underwater acoustic waves. Constructed from elements of the non-resonant circuits, this novel kind of acoustic metamaterial can operate over a wide spectrum of frequencies. Acoustic cloaking with parameter specifications can be manufactured by using acoustic circuit transmission equations, which can be found in Fig. 2(a), and is composed of a subwavelength unit cell. Additionally, the spatially varying parameter profile can be acquired by adjusting the geometric parameters of the related metamaterial structures, as presented in Fig. 2(a). The experiment demonstrates that the existence of the steel cylinder in the tank produced massive wave deflection and scatter, as shown in the left panel of the upper part of Fig. 2(b). The use of metamaterial cloaking for the steel cylinder encompassment, as displayed in Fig. 2(a), enabled the restoration of wave trajectory behind the cloak with tiny deformation in the cylindrical wavefronts. In this way, the hidden cylinder and cloaking became invisible under the hydrophone. To reveal the broadband performances of the metamaterial cloaking, the acoustic pressure field distributions both with and without cloaking are presented in the middle and right panels in the bottom part of Fig. 2(b).

### Pentamode metamaterials

Pentamode metamaterials (PMs) refer to fluid-like metamaterials with an extremely low shear modulus and are able to offer a highly anisotropic bulk modulus based on interconnected microstructures, as first proposed by Milton and Cherkaev [57] in 1995. Numerical calculations using three-dimensional microstructures have been used to demonstrate that the bulk modulus to shear modulus ratio can realistically be approximately 1000 [58]. Pentamode metamaterials can be used for acoustic wavefront manipulation and underwater acoustic bending [59,60]. Acoustic cloaking has been realized through the microstructure design of a lattice pentamode metamaterial [61,62]. A potential strategy aims to explore the anisotropic modulus instead of the density, which can be fabricated by annular underwater acoustic cloaking designed from graded solid microstructures. Additionally, the annular cloak, which was machined from aluminum cloaking via advanced high-precision electrical discharge machining, is displayed in Fig. 2(c). Along the θ direction, the assembled sectors of cells amounted to 50, and there were five graded PM unit cells for each of the sectors along the circumferential direction. As the initial step of the cloaking design, the density ρ and principal moduli *k*θ and *kr* were derived by applying a transformation theory with an optimization algorithm, and the realization of necessary material parameters was accomplished through geometric calibration of the PM unit cells. The upper portion of Fig. 2(d) presents the snapshots of pressure field measurements for the forward zone, respectively corresponding to the cloaked, uncloaked and reference cases. The backward region as shown in the middle of Fig. 2(d) illustrates the cloaked, reference and uncloaked cases in both forward and backward sound pressure fields. Obviously, the cloaked case exhibits less backscattering than the uncloaked case. However, the reflected wave for the uncloaked case indicates that the steel cylinder target can be easily detected without metamaterial cloaking.

### Underwater carpet cloaking

Another approach to underwater acoustic invisibility is underwater acoustic carpet cloaking. Broadband carpet cloaking in optical frequencies was first proposed by Jensen and Pendry [63]. However, owing to the difficulty in obtaining a unit cell with the required parameters, underwater acoustic carpet cloaking has not been achieved experimentally. Fortunately, transformation acoustics exhibits extraordinary performance in forming composites with elastic tensors. One realization of underwater carpet cloaking was evaluated experimentally through an underwater acoustic experiment [64]. The proposed carpet cloaking method demonstrates its ability to control underwater acoustic waves on a deep subwavelength scale. The 3D underwater acoustic carpet cloaking can be acquired via steel stacked layers embedded by water, and the required parameters include the anisotropic mass density as well as the isotropic bulk modulus [44]. To reduce the difficulties of material preparation caused by the anisotropic mass density, quasi-isotropic underwater acoustic carpet cloaking with a 2D version of the pentamode lattice was designed based on a quasi-conformal transformation [65]. The performance of the designed pentamode carpet cloaking (PMC) via a quasi-conformal transformation was assessed by a full-wave numerical simulation, which showed that the latticed PMC exerted a nearly perfect impact in concealing the bump in a broadband frequency domain. Given that the physical and virtual spaces can be depicted by complex coordinates *w* = *u* + *iv* and *z* = *x* + *iy*, respectively, the conformal transformation optics can construct the correlation between these two spaces as


**Figure 3.**
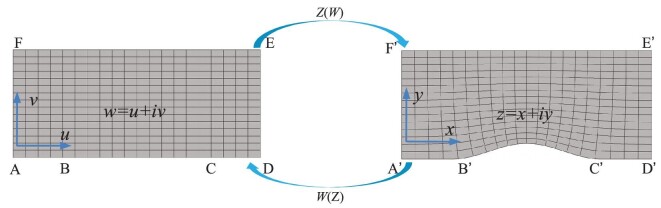
The schematic diagram of quasi-conformal mapping from the regulated rectangular space into the transformed curved space, which was adapted with permission from Sun *et al.* [63], Appl Phys Lett 114, 094101 (2019). Copyright 2019, AIP Publishing.

(5) \begin{eqnarray*} {n_z} = {n_w}\bigg | {\frac{{dw}}{{dz}}} \bigg | = {n_w}\sqrt{\frac{1}{{\det J}}}, \end{eqnarray*}

where *n_z_* and *n_w_* refer to the acoustic refractive index in both original space and transformation space, respectively, and *J* denotes the transformation tensor. The above transformation tensor can be obtained by mapping the transformation function between physical and virtual spaces, *w*(*z*). The sound wave propagation in two different spaces with homogeneous medium should follow the corresponding Helmholtz equation. As a result, the form invariance of the Helmholtz equation is also employed in acoustics and the correlation in the above equation can also be maintained in acoustics. Conformal mapping simplifies the material anisotropic parameters, which gives a direct metamaterial design scheme for an isotropic medium. Fig. 3 presents the original and transformed spaces of carpet cloaking.

The refractive index *n_w_* is indicated by the virtual space ABCDEF, whereas transformed media-filled carpet cloaking with refractive index *n_z_* is indicated by the transformed space A’B’C’D’E’F’. Water is used as the background medium, whose density ρ_0_ is *p*_0_, bulk modulus is *K*_0_ and refractive index *n_w_* = *n*_0_. Transformation matrix *J* is utilized for the determination of *n_z_*. However, it is still extremely difficult to find an appropriate mapping *z*(*w*) for the proposed carpet cloaking. An adequate measure in this scenario is the quasi-conformal transformation (QCT). Through numerical solving of inverse Laplace equations under preset boundary conditions, the inverse mapping *w*(*z*) can be computed, which is a strategy for realizing QCT. The QCT theory holds that in the mapping *w*(*z*), the Cauchy-Riemann conditions need to be fulfilled, i.e.


(6) \begin{eqnarray*} \frac{{\partial u}}{{\partial x}} = \frac{{\partial v}}{{\partial y}}, \end{eqnarray*}

(7) \begin{eqnarray*} \frac{{\partial u}}{{\partial y}} = - \frac{{\partial v}}{{\partial x}}, \end{eqnarray*}

which can lead to the Laplace equations


(8) \begin{eqnarray*} {\nabla ^2}v = 0, \end{eqnarray*}

(9) \begin{eqnarray*} {\nabla ^2}u = 0. \end{eqnarray*}

In solving the Laplace equations, considering the Dirichlet and Neumann boundary conditions


(10) \begin{eqnarray*} \mathop n\limits ^ \rightarrow \cdot \mathop \nabla \limits ^ \rightarrow u(x,y){|_{A^{\prime }B^{\prime }C^{\prime }D^{\prime },E^{\prime }F^{\prime }}} = 0, \end{eqnarray*}

(11) \begin{eqnarray*} v(x,y){|_{A^{\prime }B^{\prime }C^{\prime }D^{\prime },E^{\prime }F^{\prime }}} = y, \end{eqnarray*}

(12) \begin{eqnarray*} \mathop n\limits ^\rightarrow \cdot \mathop \nabla \limits ^\rightarrow v(x,y){|_{F^{\prime }A^{\prime },D^{\prime }C^{\prime }}} = 0, \end{eqnarray*}

(13) \begin{eqnarray*} u(x,y){|_{F^{\prime }A^{\prime },D^{\prime }E^{\prime }}} = x, \end{eqnarray*}

the inverse Jacobian matrix *A* = *J*^−1^ can be written as


(14) \begin{eqnarray*} A = {J^{ - 1}} = {\left(\begin{array}{cc}{{\partial u}}/{{\partial x}}&{{{\partial u}}/{{\partial y}}}\\ {{{\partial v}}/{{\partial x}}}&{{{\partial v}}/{{\partial y}}} \end{array}\right)}. \end{eqnarray*}

Slight anisotropy can be generated due to the existence of cross terms in the transformation tensor. Therefore, the transformation can be considered to be a quasi-conformal process. In addition, the impedance-matching condition should be considered under the process of QCT, with the effective density and bulk modulus given by as


(15) \begin{eqnarray*} \rho = \frac{{{\rho _0}{c_0}}}{c} = {\rho _0}n = {\rho _0}\sqrt{\frac{1}{{\det J}}}, \end{eqnarray*}

(16) \begin{eqnarray*} K = \rho {c^2} = \frac{{{K_0}}}{n} = {K_0}\sqrt{\det J}. \end{eqnarray*}

**Figure 4.**
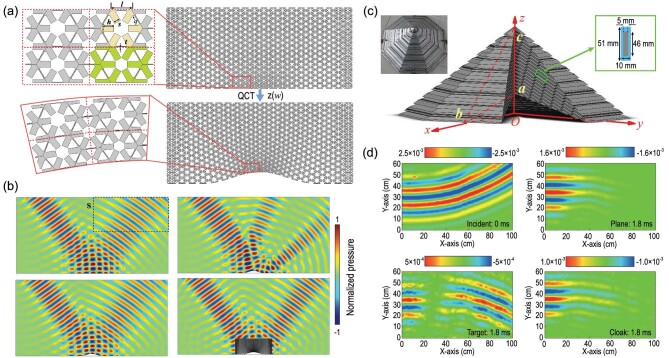
(a) Micromorphological structure of 2D PMs, where yellow indicates its basic unit cell and green indicates its brick. The deformed PM bricks are obtained by QCT from the regular PM bricks. (b) Acoustic pressure fields for the rigid plane, rigid scatter, latticed PMC and theoretical cloaking at a frequency of 15 kHz incident at 55°. (c) Schematic illustration of the 3D UACC. An enlarged view of a representative unit cell is displayed in the green frame, while the left inset in (c) presents an actual image of the 3D UACC sample. (d) The 10 kHz measurements of the acoustic pressure field in the rectangle frame. The 0 ms value of the incident pressure field, and the 1.8 ms values of the scattered pressure fields from the soft plane, soft target as well as cloaked soft target. Panels (a) and (b) are modified based on the permission from Sun *et al.*, Appl Phys Lett 114, 094101 (2019). Copyright 2019, AIP Publishing. Panels (c) and (d) are modified with permission from Bi *et al.*, Appl Phys Lett 112, 223502 (2018). Copyright 2018, AIP Publishing.

In this work, QCT was adopted for deforming the pentamode metamaterial array into the appropriate shape, with the purpose of forming the desired carpet cloaking. Because QCT can be nearly conformal, the angle of the metamaterial line along different directions is almost constant, as shown in Fig. 4(a). The performance of the latticed pentamode carpet cloaking was then simulated, and the simulation results are presented in Fig. 4(b). The empty acoustic field case and the scattering acoustic field with the rigid ground plane are shown in the top left and top right panels, respectively. By introducing carpet cloaking with theoretical parameters and pentamode metamaterials by QCT, the acoustic pressure fields are illustrated in the bottom left and bottom right panels of Fig. 4(b), respectively.

### Three-dimensional underwater acoustic carpet cloaking

Subsequently, 3D underwater acoustic carpet cloaking (UACC) was designed and proposed [44]. Artificial structures with steel strip arrays were assembled to obtain the required acoustic parameters. According to observations, the triangular pyramid cross section comprises periodical steel strips in a rectangular lattice. The steel strips were 46 mm wide and 5 mm thick, while the lattice dimensions were 51 × 10 mm^2^ (Fig. 4(c)). Through the space position-dependent length alteration, these strips constitute a triangular pyramid. Figure 4(d) details the incident acoustic field and scattering field. After originating from the upper left corner, the omnidirectional wave disperses, propagates into the bottom area and 1.8 ms later is reflected by the soft target and plane to exhibit a bottom-to-top propagation. Following the target coverage with 3D UACC, contrastively, the scattering acoustic wave resumes the direction of backscattering. The scattering wave exhibits amplitude and phase that closely approximate those of the soft plane.

## UNDERWATER BEAM FORMATION

### Effective medium theory of underwater metamaterials

Considering the liquid-solid metamaterials, isotropic solid cylinders in water can achieve the effective parameters of the designed sound speed and mass density profiles. The original sound speed section and transformed sections can be divided into several discrete regions by solid cylinders of different sizes, and the effective parameters can be described as [66]


(17) \begin{eqnarray*} {\rho ^*}=\varphi {\rho _1}+(1-\varphi ){\rho _0}, \end{eqnarray*}

(18) \begin{eqnarray*} {c_L}^*=\sqrt{\frac{{{\lambda ^*}+2{G^*}}}{{{\rho ^*}}}}, \end{eqnarray*}

(19) \begin{eqnarray*} {c_T}^*=\sqrt{\frac{{{G^*}}}{{{\rho ^*}}}}, \end{eqnarray*}

where ρ*, $c_L^*$ and $c_T^*$ denote the effective density and speeds of the longitudinal and transverse waves, respectively. The effective Lamé coefficient and shear modulus can be approximated by the equations


(20) \begin{eqnarray*} {\lambda ^*}=\frac{{(M-1){G^*}+(M+1){G_0}+{\lambda _0}}}{{1-M}}, \end{eqnarray*}

(21) \begin{eqnarray*} {G^*}=\frac{{{G_1}(1+\varphi )+{G_0}(1-\varphi )}}{{{G_1}(1-\varphi )+{G_0}(1+\varphi )}}{G_0}, \end{eqnarray*}

(22) \begin{eqnarray*} M=\frac{{[{\lambda _1}+{G_1}-({\lambda _0}+{G_0})]\varphi }}{{{\lambda _1}+{G_1}+{G_0}}}. \end{eqnarray*}

The densities of the substrates used were ρ_0_ and ρ_1_, respectively. The Lamé coefficients of the substrate and solid cylinders used were λ_0_ and λ_1_, respectively. The shear moduli of the substrate and solid cylinders were *G*_0_ and *G*_1_, respectively.

### Underwater biomimetic projector

**Figure 5.**
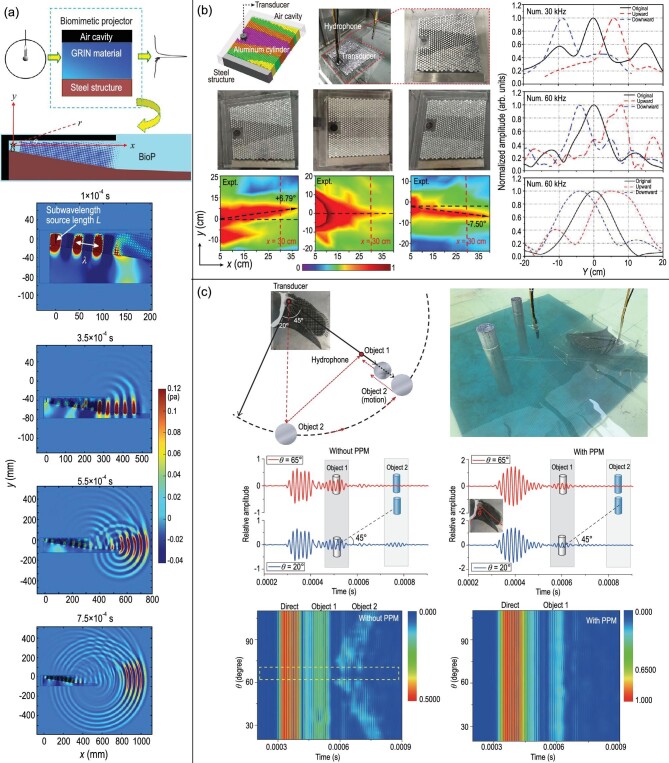
(a) Schematic representation of the biomimetic projector (BioP), in which the subwavelength source was converted into the directivity beam. (b) Four different filling fractions to approach the upward acoustic model were obtained by BCT. The different colors represent the corresponding radii of the aluminum cylinders, steel structure, air cavity and transducer, respectively. An omnidirectional transducer and hydrophone were used for underwater metamaterial measurement. Photograph of the three fabricated metamaterial models. Distribution of acoustic pressure measurements for the upward (main lobe angle +6.79°), original (main lobe angle 0°) and downward (main lobe angle −7.50°) models. (c) Application of directional target detection with the physics-based porpoise model (PPM) device. Photograph describing the underwater detection of the target. The underwater detection was accomplished utilizing object 1 and its interfering object 2. Distribution of pressures respecting θ of the system with PPM device. Panel (a) was modified with permission from Zhang *et al.*, Appl Phys Lett 105, 123502 (2014). Copyright 2014, AIP Publishing. Panel (b) was modified with permission from Dong *et al.*, Phys Rev Appl 13, 024002 (2020). Copyright 2020, American Physical Society. Panel (c) was modified with permission from Dong *et al.*, Nat Rev Phys 6, 921–8 (2019). Copyright 2019, Oxford University Press.

Moreover, acoustics sensing and communication benefit from fascinating designs in nature [67–69]. Recently, a structure biomimicking the biosonar system of the Yangtze finless porpoise was developed based on computed tomography, which converted an air cavity (from air sac mimicking), gradient-index material (for melon mimicking) and steel exterior (for skull mimicking). Compared to the subwavelength source absent from the biomimetic structure, about a three-fold higher main lobe pressure was attained. A biomimetic projector (BioP) was proposed to manipulate the omnidirectional wave produced by a subwavelength source into a directivity beam, which was inspired by the porpoise’s multi-phase forehead structure [70], as illustrated in Fig. 5(a). Finite element simulations were conducted with the aim to simulate the directional acoustic beam spreading with time by BioP as shown in the right panel of Fig. 5(a). At a propagation time of *t* = 1 × 10^−4^ s, the subwavelength (λ > *L*) diffraction enabled wave propagation along the GRIN material. Nevertheless, with the wave reaching the BioP, a complicated multi-phase structure produces the directivity beam, which can be found on the right side of Fig. 5(a). The acoustic beam is prone to bending by the GRIN material, whose acoustic velocity is lowest in the inner core. Acoustic impedances of the air cavity and steel differed greatly from that of water, so that in the biomimetic system, prominent interfacial waves are generated, as well as reflections and scattering of waves. Probably, the Scholte waves on the surface of the steel structure and GRIN material are implicated in the propagation of waves within the BioP.

### Bioinspired transformation acoustics

Because of the intricate process of deformation, the distributions of acoustic velocities can be uncertain. To achieve arbitrary acoustic directional manipulation, identifying the relationship between the shape deformation and acoustic parameter distribution is required. Hence, the theory of bioinspired conformal mappings has been proposed for understanding how geometric deformation induces dynamic tunable processes of acoustic parameters. Based on the above discussion, a novel strategy of bioinspired conformal transformation (BCT) for collimation emission and acoustic steering is proposed, as illustrated in Fig. 5(b). The developed steering and collimation models comprise three structures, namely, an air cavity for air sac biomimicking, a gradient-index distribution metamaterial for forehead tissue biomimicking and steel for skull biomimicking. Further numerical simulation and experimental verification were accomplished on the forehead shape deformations and corresponding control of the acoustic beam [45]. Applications have been evaluated for underwater target detection and ranging. The first step is to obtain conformal mapping based on the Laplacian equation. This conformal transformation involves changing the rectangular field to a non-rectangular field. This corresponds to the forehead deformation of toothed whales such as pygmy sperm whales and dolphins.

Subsequently, an underwater bioinspired acoustic model based on the above effective medium theory can be designed and the effective sound speed of an aluminum cylinder embedded in background water can be obtained. An assembly drawing of the upward acoustic steering model is given in Fig. 5(b, upper left). A photograph of the assembled original acoustic model is given in Fig. 5(b, upper middle). The bioinspired acoustic models are composed of three components: (1) the maxilla, fabricated from steel using laser cutting; (2) the air component, consisting of a hollow cavity; (3) the metamaterial unit cells, composed of aluminum cylinders. The experiment was conducted in a water tank with a scale of 1 × 1.2 × 1.5 m^3^, and the 3D acoustic platform was connected to the outer edge of the water tank. A moving motor was used to drive the movable mechanical arm with an attached broadband hydrophone to obtain sound pressure statistics (Fig. 5(b, upper middle and upper right)). The lower row of Fig. 5(b) illustrates the measured sound pressure distributions of the bioinspired models. Additionally, the experimental findings were in good agreement with the numerical results.

### Directional emission and target detection

Artificial metamaterials and animal biosonar have the potential to overcome size-wavelength limitations. According to the latest research, toothed whales are capable of generating underwater directional beams and adjusting their widths through forehead compression [71]. As demonstrated by this artificial structure, directional control over an acoustic beam is achievable [71,72]. As displayed in Fig. 5(c), the angle of biomimetic emission at subwavelength scale and the width of directional beams have been validated via experiments. Prior reported acoustic manipulations by a subwavelength corrugated surface and collimation via a decorated plate have been demonstrated in various studies [73,74]. The physics-based porpoise model (PPM) proposed using a hybrid metamaterial consists of a programming microstructure. For the underwater acoustic application of acoustic directional detection, target recognition is enhanced by the PPM. Aluminum object 1, whose location was along the axis of the main beam, was arranged 1 m away from the source. Object 2, the second aluminum target, was arranged 10 cm to the rear of object 1 for interfering purposes. The hydrophone was placed between the transducer and object 1, at a distance of 10 cm from the object. The acoustic absorbing material is capable of receiving cleanly scattered and direct signals from both of the objects. Figure 5(c) displays the pressure measurement comparisons between the detector with and without the PPM device. In the case when the scope of the beam angle is as shown inside of the dashed line in the bottom portion of Fig. 5(c), both systems, either with or without the PPM device, can detect the echo from object 1. In contrast, in the case when θ is outside such a beam width, only a scattered echo from object 1 was received by the detector with the PPM device, while the scattered echoes from both objects 1 and 2 were received by the detector devoid of the PPM device. Given the PPM’s directional trait, the noise from zones outside the main beamwidth was removed and the signal from the interference target was diminished. Therefore, the acoustic emission process undergone by the PPM device resembles that of porpoise biosonar. The PPM is capable of improving the target detectability and signal-noise ratio; it is profoundly applicable to acoustic detection in an underwater context.

## UNDERWATER METASURFACES AND PHASE ENGINEERING

Alongside the exploration for enhancing wave interplays with matter and achieving wave manipulation with minimum space utilization, research on acoustic metasurfaces has emerged. Metasurfaces are a class of wavefront-shaping devices, whose thicknesses are considerably thinner than the wavelength. In recent years, because of their capability of modulating the phase and amplitude, and achieving near-perfect absorption with subwavelength dimensions, acoustic metasurfaces that offer phase modulation have attracted significant interest. Metasurfaces can be classified into three types on the basis of the structures of the coiling-up space [18,19,75–77], Helmholtz resonators [78–80] and membranes [81–84]. In comparison with their applications in air, it is more difficult to create a completely hard boundary condition assumption to support the realization of underwater acoustic wave manipulations. Nevertheless, acoustic phased arrays can produce an arbitrary acoustic wavefront by dynamically modulating the phase delay in every transducer element. In recent years, the emergence of digital materials has presented miniaturized and programmable design methods in microwaves, optics and acoustics. Phase encoding technology in phase engineering can achieve complex wavefront shaping; thus, it is widely used in reflected waves [85], transmitted waves [75] and acoustic beam generators [78]. Phase encoding can be achieved through the resonant phase shift of a helical microstructure [86], or generalized Snell’s law [87]. Labyrinth and spiral structures in coiling-up-space acoustics are useful for changing the sound path to control the transmission phase [88,89].

### Underwater subwavelength asymmetric transmission and acoustic switches

Subwavelength asymmetric transmission devices allowing one-direction acoustic wave propagation have drawn much attention due to their potential applications in subwavelength control. However, most of the proposed designs are considerably larger than one wavelength, which is not practical for general applications requiring miniaturization at low frequencies. The underwater subwavelength asymmetric acoustic transmission (SAAT) device was developed on the basis of solid-fluid interactions [49].

The SAAT comprises the periodic rectangular grating responsible for the conversion of the wavefront and a 1D superlattice responsible for the forbidden transmission at low frequencies. Despite mere 0.6λ and 0.128λ (with λ the wavelength) constants for the SAAT structure and superlattice, respectively, it can achieve an extremely high rectifying efficiency of over 10^8^. Apart from overcoming the size-wavelength limitation, this SAAT design also enables unidirectional transmission of low-frequency acoustic waves, making it ideal for integrated acoustic device miniaturization during the unidirectional transmission of signals. Existing phononic-crystal-based acoustic asymmetric transmissions have a fundamental size-wavelength limitation. For instance, multiple scattering is allowed due to the considerably larger device size *L* compared to the acoustic wavelength λ, which is thus inapplicable to the devices having subwavelength size (*L* < λ). Therefore, unidirectional subwavelength transmission is impossible through the exploitation of this principle. To miniaturize the device, the transmission structures need to be asymmetric, and their sizes should be compact and subwavelength. Remedying such a limitation necessitates the design of SAAT(*L* < λ). Then, a water tank was utilized to immerse the whole assembly. Through the forbidden transmission of the solid-fluid superlattice at low frequencies, subwavelength unidirectivity is achieved. The rectifying efficiency attained by this passive SAAT device is exceedingly high, where external active power is unnecessary.

**Figure 6.**
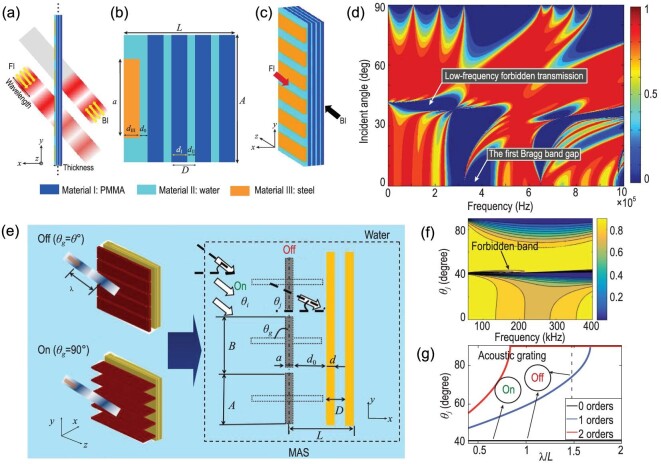
(a) Schematic illustration of the subwavelength asymmetric acoustic transmission (SAAT) device. (b) Schematic presentation of one unit cell. (c) Depiction of the 3D multi-layered structure. (d) Variation of the transmission coefficient with incident angle and frequency of the multi-layered SAAT. Arrow indicates a forbidden band under low frequencies. (e) Systematic depiction of the MAS structure. (f) Transmission coefficient variation with incident angle and frequency, where *N* = 2. (g) Correlation of normalized wavelength λ/*L* with diffraction angle. Panels (a–d) are modified based on permission from Zhang *et al.*, Phys Rev Appl 5, 034006 (2016). Copyright 2016, American Physical Society. Panels (e–g) are modified with permission from Cao *et al.*, Phys Rev Appl 13, 044019 (2020). Copyright 2020, American Physical Society.

It is clear from Fig. 6(a) that there are two major parts to the present SAAT design. A multi-layered SAAT composed of periodically arranged PMMA and water constituted the first part. The second part consisted of 1D water-immersed steel, whose arrangement was periodic along the *y* direction. Arrows in Fig. 6(a) respectively point to directions of forwarding incidence (FI) and backward incidence (BI). Figure 6(c) illustrates the complete 3D structure. In the present research, the transfer-matrix approach was employed to perform the omnidirectional transmission estimation on the solid-fluid superlattice, while stationary simulations were applied to describe the operation of the asymmetric transmission devices. Figure 6(d) shows the subwavelength transmission traits for the four-layer solid-fluid superlattice, where the transfer matrix approach is employed to determine the transmission coefficient variation with both the incident angle to superlattice and frequency. Forbidden transmission at low frequencies means that the energy is quickly attenuated upon considerable diminishment of the lattice constant compared to the wavelength, which differs from the Bragg scattering-induced band gaps, as represented by first Bragg band gap in Fig. 6(d).

Based on the above transfer-matrix method, it is necessary to evaluate the possibility of underwater acoustic switches. The mechanism of electronic switches has inspired the development of acoustic switches. Band gap opening and closing are controlled by Bragg scattering or local resonance [90]. A class of spring structural acoustic metamaterials has been proposed that utilizes a deformable structure to switch on and off sound propagation. The underlying mechanism is the existence of a band gap in the undeformed configuration. When the helical line is stretched axially, the band gap can be controlled to form a passband to allow sound propagation, which allows it to be controlled freely. Babaee *et al.* [91] proposed a quasi-acoustic material with deformable elastic spirals to turn sound propagation on and off. However, energy bands have only been studied in two dimensions, which is quite limited. It is a great challenge to use the above-mentioned energy band theory in an underwater environment. Metasurfaces have the significant advantage of thin layers and have been used in areas such as beam control, tunable permeable lenses and acoustic stealth. However, it is difficult to examine underwater acoustic switches because of the influence of acoustic structural coupling. A free-controlled underwater metasurface acoustic switch structure (MAS) has been proposed recently [48]. It consists of an acoustic grating sequence and double-layer PMMA (Fig. 6(e)). The principle is to use acoustic grating to control diffraction and one-dimensional superlattice acoustic band gap transmission control to overcome underwater shortcomings and achieve an acoustic mode conversion (Figs. 6(f)–(g)).

### Underwater acoustic vortex beam

**Figure 7.**
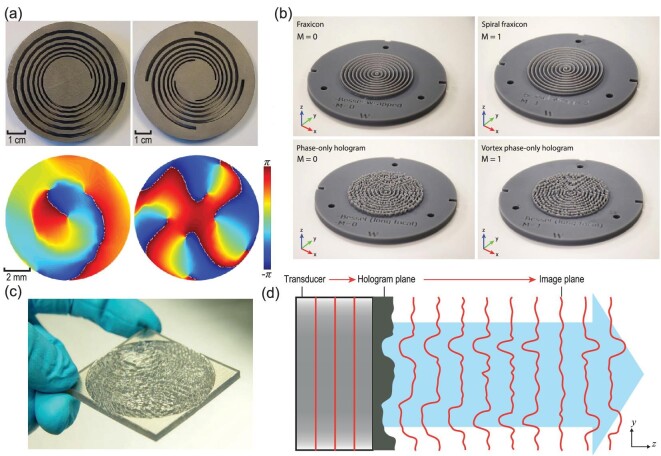
Actual images of the one- and four-armed MACS samples. The right side of (a) reveals the schematic of the experimental setup. The left bottom portion of (a) details the 425 kHz phase distribution measurements of the one- and four-armed MACSs at the cross section *z* = 2.5λ. (b) Fraxicon for the zeroth-order Bessel beam corresponding to *M* = 0, spiral fraxicon (*M* = 0 and *M* = 1) (vortex) and phase-only holographic lenses for flat-intensity Bessel beams (*M* = 0 and *M* = 1) (vortex). (c) Three-dimensional printed hologram. For every pixel, its phase delay is in proportion to its thickness. (d) The schematic represents the conversion of the emitted wavefront from a transducer to the desired phase distribution through an encoded topography of an acoustic hologram surface. Panel (a) was modified with permission from Jiang *et al.*, Appl Phys Lett 108, 203501 (2016). Copyright 2016, AIP Publishing. Panel (b) was modified based on permission from Jiménez-Gambín *et al.* [92], Sci Rep 9, 1–13 (2019). Copyright 2019, Macmillan Publishers Ltd. Panel (c) and (d) were modified with permission from Melde *et al.*, Nature 537, 518–22 (2016). Copyright 2016, Macmillan Publishers Ltd.

In recent decades, acoustic vortex beams with orbital angular momentum (OAM) have revealed significance [50,89,93]. One representative feature of acoustic vortices refers to the helical phase distribution with the phase angular θ in *e*^*im*θ^. The integer *m* denotes the topological charge, showing consistency with the order of the vortex, determining the acoustic phase accumulation within a full circle near the propagation direction. Principally, acoustic vortices can avoid the complex acoustic phased arrays, which are also known as active control. Nonetheless, there are several shortcomings with the active method, which make it less practical. In technical terms, the conventional active method attains the suitable amplitude and phase by electronically controlling individual pixels in the transducer array, which is cost ineffective. The acoustic transducer or loudspeaker employed as the array’s individual element, however, has finite dimensions analogous to the acoustic wavelength at all times. Because of the limited discretization precision, the applicability of miniaturization is drastically restricted. There have been reports of multi-arm coiling slits (MACSs) in analytical designs, numerical simulations as well as experimental realizations. Through the wave phase and amplitude modulation in the diffraction field, stable twisted acoustic vortices are created by the MACS-based generator. For the entire cases of one- to four-armed coiling slits, a long-distance expansion of the topological charge’s sustenance and stability is achievable along the direction of propagation. Notably, since MACSs are devoid of resonating components, they possess intrinsic broadband functionality, a practically significant trait. For validation of the proposed MACS-based production of stabled broadband acoustic vortices, experiments have been designed and implemented. The photographs in Fig. 7(a) are from the 3D metal-printed samples of the one- and four-armed coiling slits. The material of the samples was set as stainless steel, which could be used as the rigid material in water. The underwater acoustic experimental results for the one- and four-armed vortex structures are shown in the bottom part of Fig. 7(a).

### Underwater Bessel beam

Bessel functions refer to one typical solution of the acoustic Helmholtz equation, that the beamwidth of the ideal Bessel beam is close to the diffraction-limited beamwidth and shows an outstanding non-diffraction transmission property. Bessel beams were initially proposed by Durnlin [94] in 1987 and have been extensively applied in both optics and acoustics. A recent study illustrated an easy approach to generating zero- and high-order Bessel beams with non-diffraction properties along the propagation axis by phase-only holography. Additionally, phase-only acoustic holography can be realized by phase and amplitude encoding. The experimental test was carried out at the ultrasonic frequency ranges with a phase-modulated acoustic hologram plate of diameter 2*a* = 50 mm and non-diffraction field *F* = 40λ underwater. Additionally, a fraxicon was also manufactured for comparison with the hologram plate. Both the zeroth- and first-order Bessel beams were taken into consideration. The fabricated Fraxicon lens, spiral fraxicon lens, phase-only hologram lens and vortex phase-only hologram lens are shown in Fig. 7(b).

### Underwater acoustic hologram

Holographic techniques set the foundation in applications, including high-density data storage and spatial control of intricate optical [95] or acoustic fields [96,97]. In ultrasound applications, acoustic transducers produce a phase profile by discretely and independently driven ultrasound sources, which are referred to as phased arrays. By controlling the incident phase distributions in the emitter plane, the phased array can produce complex acoustic beams, including vortex beams [50,89,93], self-accelerating beams [98] and collimation beams [99], and can achieve super-resolution imaging under sub-diffraction-limited conditions [100,101]. Compared to traditional phased arrays, a metasurface provides a miniaturized, compact and effective passive strategy for independent phase and amplitude control. Each unit cell on a metasurface can generate a gradient phase shift by utilizing resonator-based coiling-up space structures. Computational acoustic holography can reconstruct the phase information of the hologram plane based on the amplitude information of the target imaging plane, which can avoid complex phase accumulation calculations. Because of the shortage of large impedance differences between water and solid structures, the underwater acoustic hologram has yet to be widely investigated. In recent years, a kind of 3D-printed acoustic hologram plate with diffraction-limited resolution has been fabricated for achieving arbitrary phase accumulation [51]; the printed acoustic hologram model is shown in Fig. 7(c). The phase distributions in the hologram plane can be produced using the iterative angular spectrum approach (IASA). In addition, the 3D-printed non-uniform hologram plate records the desired encoding information with increasing diffraction distance. Moreover, the designed hologram plane had been positioned in front of the transducer surface. The plane wave generated by an ultrasonic piezoelectric transducer can be converted to the expected sound pressure field distributions using IASA. By experimentally measuring the sound pressure distribution on the imaging plane by hydrophone, the image plane reconstructs the target image accordingly. The reconstructed degree of freedom of the new acoustic hologram technique proves to be 200 times higher when compared with those of commercial medical ultrasonic transducer arrays. This new acoustic hologram technology is capable of recording more information within the limited space, providing higher acoustic transmission energy and offering a possibility for higher ultrasonic imaging resolutions.

## UNDERWATER TOPOLOGICAL ACOUSTICS

### Topological edge states

**Figure 8.**
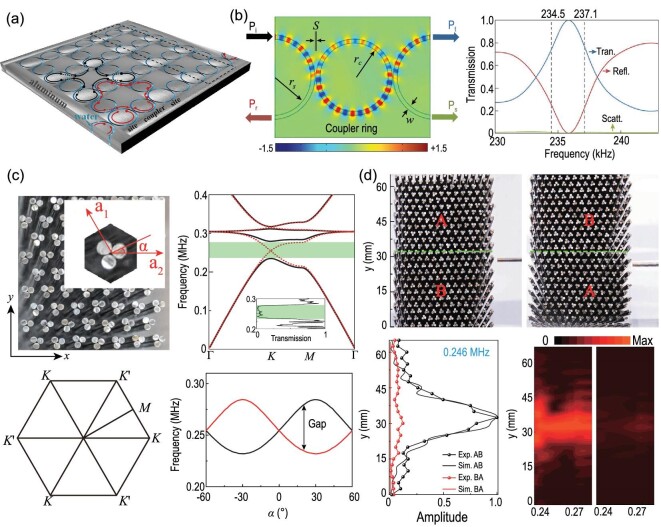
(a) Schematic illustration of a square lattice comprising water annulation embedded in an aluminum background. The arrow and opposite arrow respectively indicate the clockwise and counterclockwise circulating acoustic modes. The supercell used for computing the projected band structure is displayed in the dashed rectangle box. (b) Configurations for the site ring and coupler ring with respective inner radii of *r_s_* (19 mm) and *r_c_* (17 mm), and identical widths ω (2 mm). The frequency scope estimate for gapless edge states is indicated by the dashed line. (c) An image with the upper panel depicting the water-immersed SC made of metamolecules in a triangular lattice array. The inset portrays its unit-cell geometry, while the lower panel depicts the relevant first Brillouin zone. Every metamolecule is formed by three steel cylinders packed tightly. A Gaussian beam is directed toward the interface from the left-hand side of the sample. Panels (a) and (b) were adapted with permission from He *et al.*, Appl Phys Lett 108, 031904 (2016). Copyright 2016, AIP Publishing. Panels (c) and (d) were adapted with permission from Shen *et al.* [113], Appl Phys Lett 114, 023501 (2019). Copyright 2019, AIP Publishing.

Topological edge states exert a vital function in overcoming the limitations of traditional acoustic disorders. Researchers have been working on the realization of 2D acoustic topological insulators [33,102,103] and related edge modes protected by symmetry. Recently, defect states of artificial topological metamaterials have attracted extensive interest from researchers. Topological concepts are universal and include electromagnetic [104,105], optical [106,107] and acoustic [33,103] concepts. However, all these studies have focused on artificial structures in elastic waves [102,108–110] and airborne acoustics [33,103,111,112]. Owing to the significant influence of the water medium on the acoustic mode conversion of structures, there are few studies on underwater topological acoustics. The coupling ring joint structure has been designed in an underwater environment [28] in which researchers use lattice design in the coupling ring resonator edge of the topology. A single ring of two decoupled modes can approximate the electron spin. This design was inspired by an acoustic coupling ring resonator array without destroying the time-reversal symmetry, realizing the phonon quantum spin Hall effect. Figure 8(a) shows a schematic of the aluminum background with lower acoustic impedance for clockwise and counterclockwise circulating acoustic modes. The sound waves are coupled through the structure, and the mode in the coupler ring is opposite to the mode in the position ring owing to directional coupling without any acoustic scattering. These two models simulate the pseudospin of an electron and produce the opposite pseudospin-dependent effective vector potential. Without considering any loss, matrix


(23) \begin{eqnarray*} S = {e^{i\chi }} {\left[\begin{array}{cc}\sin \theta &{\cos \theta }\\ {i\cos \theta }&{\sin \theta } \end{array}\right]}, \end{eqnarray*}

where θ is from 0 to 2π and χ represents the total phase coefficient [114,115]. The calculated energy bands are shown in Fig. 8(b). The analysis shows that the underwater acoustic topological structure can reverse two back propagating pseudospins. By controlling the coupling strength of the ring cavity, the energy band of the topological phonon state could be designed as a gap-free boundary. In a ring resonator, two pseudospins cause sound to propagate along different edges.

### Topological valley transport

Thus far, studies about acoustic topological insulators mainly concentrated on elastic wave metamaterials and airborne acoustic metamaterials. Water refers to a vital sound medium, and underwater acoustics exert a critical function in underwater communication, positioning and telemetry. Additionally, the development of underwater acoustic topological insulators is of great interest. Recently, valley-projected edge modes were observed in underwater topological experiments. As shown in Fig. 8(c), the cells are hexagonal lattices composed of three identical structures. The valley-projected edge states of underwater sounds were studied. The properties of the sonic crystal (SC) depend on the arrangement of the structure of the three units (Fig. 8(c)). When α = *n*π/3, the SC exhibits a quadratic transformation at the corner of the first Brillouin region. In other cases, the omnidirectional band gap opens because of the mismatch between the lattice and ternary element structure. This was confirmed by the experimental transmission spectrum (Fig. 8(c)). If α is the opposite then there are different acoustic responses. Based on the experiment in Fig. 8(d), in the entire gap region, the coupling efficiency of the two different arrangements of local symmetry and antisymmetric eigenstates is different, which is related to the incident sound wave. The topological valley transport of sound can be designed for different functional devices. Its advantages include simple fabrication and a wide working band. It has wide application prospects in underwater environments, including underwater sound signal processing and ocean noise control.

## UNDERWATER ACOUSTIC METAMATERIAL ABSORBER

The low-frequency underwater acoustic absorption has drawn considerable interest, particularly in marine noise management and anechoic pools used for testing. Compared with airborne acoustic materials, the wavelength of sound waves in water is approximately five times the wavelength of air at the same frequency. Therefore, low-frequency sounds in water are more difficult to dissipate than those in air. However, the loss of sound waves in water is much smaller than that in air, and the dissipation of the background medium cannot be used for sound absorption. As underwater acoustic absorption materials, their characteristic impedance is required to match that of water to minimize sound reflection at the surface. Based on this, the absorption materials require a high loss factor so that most of the incident sound waves can be effectively absorbed. To reduce impedance mismatch, traditional underwater acoustic anechoic tiles are shaped into wavelength-scale pyramids with the aim of minimizing reflection, which is expressed to be the gradient impedance index strategy. However, for homogeneous materials, the internal loss factor is determined by the characteristic impedance; the larger the acoustic absorption coefficient, the larger the reflection coefficient. Compared with traditional sound absorption materials, various new absorption mechanisms of underwater acoustic metamaterial absorbers [116] were brought in, such as the viscous dissipation of the micro-perforated panel [117], Fabry-Pérot resonance [118], quasi-Helmholtz resonance [119], local resonant phononic crystals [120–122] and coupled cavity resonance [123]. However, by introducing the theoretical model of sound absorption by micro-lattices and porous materials [124], it is demonstrated that effective sound absorption is dependent on micro-/macro-structured materials where acoustic energy can be absorbed via two main mechanisms: viscous losses near the solid surface and heat conducted along the solids. Therefore, materials with larger micro-air-solid interface regions, such as porous materials [125], generally have larger loss coefficients [126].

### Viscothermal effects in acoustic metamaterials

Over the past decade, there have been development and experimental demonstrations of artificial structures with negative metamaterial behavior [127–129]. For the solid or rigid structure-based negative metamaterials, their solid-fluid interface inevitably has viscous and thermal boundary layers, which is a critical issue regarding their practical functionality since unignorable losses can be incurred. Negative index behavior is perhaps suppressed by these losses, which is not taken into account during the design. Several recent papers have explored the boundary-layer effects in metamaterials. For instance, considerably prominent losses in labyrinthine metamaterial structures were demonstrated by Frenzel *et al.* [130], suggesting their fascinating potential as all-angle and broadband subwavelength sound absorption. Guild *et al.* [131] presented the broadband non-uniform metamaterial absorbers based on the viscous effects of sonic crystals with different volume fractions. Moleron *et al.* [132] revealed that viscous effects are capable of fully avoiding the excitation of Fabry-Pérot resonances. Recently, the influence of viscothermal effects in acoustic solid metamaterials with negative acoustic parameters has been demonstrated [133]. Ibarias *et al.* [134] introduced a theory of homogenization for the viscosity of phononic crystal, showing that the acoustic absorption in homogeneous cylindrical units in water increases up to five orders higher than decay in homogeneous water. Therefore, the existence of viscothermal losses in periodic metamaterial structures should be taken into account in order to properly obtain the desired behavior.

### Local resonant underwater acoustic metamaterial absorbers

**Figure 9.**
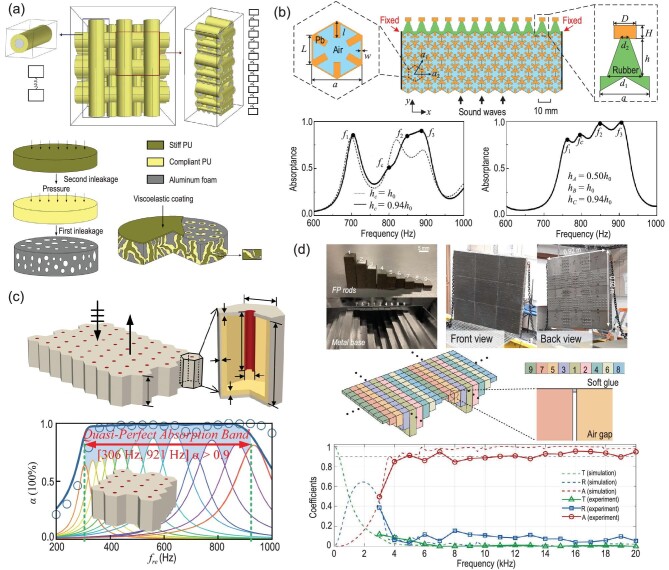
(a) Model simplification process of LRPW; three sequentially stacked sizes of soft PU-coated steel woodpile layers from perspective diagrams. Simplification of the LRPC’s 2D unit into the double-oscillator model is achievable according to the mass-spring model. The viscoelastic coating material for impedance matching with water was stiff PU. (b) Schematic illustration of the composite metasurface absorber based on an RMR-backed PM, which is sandwiched by water. (c) The geometry of the proposed metamaterial. Quasi-perfect ultra-broadband acoustic absorption for the 16-unit parallel sample. The acoustic absorption coefficients are represented by the blue graphs. (d) The integrated Fabry-Pérot (FP) resonator array indicates periodic repetition. Various colors indicate the lengths of nine Fabry-Pérot resonators, where the air gap between two adjacent rods is presented in the enlarged view. The water-facing top surface is glued thinly using waterproof soft glue. Panel (a) was modified based on permission from Jiang *et al.*, Appl Phys Lett 95, 104101 (2009) and from Jiang and Wang, J Acoust Soc Am 132, 694 (2012). Copyright 2009, American Institute of Physics and 2012, Acoustic Society of America. Panel (b) was adapted with permission from Gu *et al.*, Phys Rev Appl 16, 014021 (2021). Copyright 2021, American Physical Society. Panel (c) based on modified with permission from Duan *et al.*, Appl Phys Lett 118, 071904 (2021). Copyright 2021, AIP Publishing. Panel (d) was adapted from Qu *et al.*, Sci Adv 8, eabm4206 (2022). Copyright 2022, American Association for the Advancement of Science (AAAS).

Traditional underwater local resonant metamaterials use the principle of local resonance to generate low-frequency band gaps. Previous theoretical calculations have suggested that the maximum sound absorption occurs near the resonant frequency in a locally resonant phononic crystal (LRPC) [135,136]. This indicated that local resonant materials are good candidates for low-frequency sound absorption. However, because the band gap of a single local resonance-type metamaterial is too narrow, to achieve broadband sound absorption, Jiang *et al.* [122] proposed using a local resonance metamaterial as the phononic woodpile. As shown in Fig. 9(a), based on the theory of LPRC, several types of materials that have varying acoustic moduli can be chosen for designing the locally resonant phononic woodpile (LRPW). LRPW is composed of several varying materials; soft polyurethane (PU) has been used for coating the steel cylinders of different sizes, and hard PU that is impedance-matched underwater has been selected to be the viscoelastic coating, which allows for energy transmission through the LRPW for energy absorption. A schematic diagram of the LRPW and its fabrication process is given in Fig. 9(a). The results show that coupling between resonators of different sizes can realize the broadband sound absorption. Jiang *et al.* [120] proposed phononic glass consisting of a kind of metal-PU composite network. The physical connection of each unit cell of the LRPC can be beneficial in exciting more acoustic absorbing modes in the phononic glass. A metal skeleton connected with PU offers enhanced mechanical strength to withstand hydrostatic pressure. Additionally, Bregagne *et al.* [123] investigated the feasibility of bubble metascreens in sound energy absorption; they deduced that compact bubble rafts could be applied to be absorbing materials. Gu *et al.* [137] proposed locally resonant underwater multi-frequency acoustic metamaterials under high hydrostatic pressure, which were assessed experimentally. Because of the coupling effect of multi-frequency resonators, the low-frequency sound absorption band can be effectively expanded under relatively high hydrostatic pressures underwater.

### Underwater acoustic metasurface absorbers

In recent years, anechoic coating absorption has been enhanced by incorporating underwater acoustic metamaterials [138–140], where the chief mechanism for realizing acoustic absorption is the substrate viscoelasticity [136,141,142]. Embedding diverse inclusions, such as solid scatters [143], wooden structures [122,144] and air bubbles [145] into the polyurethane matrix rubber has been researched as substitutive measures for better enhancing the waterborne sound absorption. However, scant research efforts have been devoted to extending the operating frequency down to the kilohertz zone or acquiring a narrow and separate operating band approaching the intrinsic resonant frequency of inclusions. Since the low-frequency acoustic waves are long in wavelength, the conventional underwater sound absorbers [141,146–151] with a deep subwavelength thickness and rigid backing can hardly achieve broadband sound absorption under a frequency of 1 kHz in water. As a result, alternative schemes are necessary to develop tunable broadband absorbers for absorbing low-frequency acoustic energy to overcome the limitations of existing acoustic absorbers. An ultrathin composite metasurface was developed by Gu *et al.* [121] in a deep subwavelength thickness, which utilized the rubber-metal resonator (RMR) backed pentamode metamaterials (PMs), as displayed in Fig. 9(b). The incident acoustic wave has been converted by the PM layer into mechanical vibrations. For the sake of simplicity, it can be arranged in 2D honeycomb lattices, which have identical orthohexagonal unit cells comprising a metal framework (yellow zone) filled internally with an air medium (blue zone), as displayed in the left inset of Fig. 9(b). The composite structure parameters of RMR are determined by the effective mass-spring functions [141,152]. With the decreasing height of rubber *h_c_*, couplings between the absorptive peaks at *f*_2_ and *f*_3_ are improved because of the blue shifted *f*_2_ approaching *f*_3_, and the corresponding absorptance is shown in the bottom part of Fig. 9(b). As a demonstration, the bottom right plot of Fig. 9(b) shows considerable sound absorption near the subkilohertz frequencies, and the total thickness of the metasurface is 63 mm.

### Underwater acoustic tunable and impedance-matched absorbers

In the aeroacoustics field, substantial progress has been made in developing acoustic metamaterials based on Fabry-Pérot resonance [153–158] and Helmholtz resonance [159–167] for realizing perfect absorption at low frequencies throughout the deep subwavelength scale. Nevertheless, given the lower viscosity and higher bulk modulus of water, neither of these two resonance types can achieve valid low-frequency acoustic absorption in water. Based on former research, it can be supposed that the back layer of the metamaterials demand rigid boundary conditions. In this case, the acoustic absorption coefficient of absorbers is expressed as


(24) \begin{eqnarray*} A(\omega ) = 1 - {| R |^2} = 1 - {\bigg | {\frac{{{Z_s} - {Z_w}}}{{{Z_s} + {Z_w}}}} \bigg |^2}, \end{eqnarray*}

where the water impedance is ${Z_w} = \sqrt{{\rho _w}{B_w}}$, *R* is the reflected coefficient and *Z_s_* is the effective impedance of metamaterials. Duan *et al.* [119] proposed the quasi-Helmholtz resonance, composed of a soft-rubber coating, the cavity filling with water and an embedded hollow cylinder neck. As shown in the upper portion of Fig. 9(c), since the soft coating is composed of soft matrix and tungsten micro-particles, it provides adequate dampening and elasticity, efficient low-frequency underwater sound absorption can be realized. As shown in the lower part of Fig. 9(c), by tailoring the internal geometric parameters of each unit, the parallel sample achieved ultra-broadband acoustic quasi-perfect absorption (α > 0.9) below a frequency of 1 kHz. Good consistency is found between the theoretical forecasts and the finite element simulations. Although traditional sound absorption at low-frequency ranges has received prominent attention, nearly all the extant experimental studies carried out in the water impedance tube have been unable to reflect real performance under intricate underwater conditions. Besides, valid wave dissipation at low frequencies inside an acoustically thin sample is impeded by the rather low acoustic energy density of classic underwater materials.

Recently, Qu *et al.* [118] presented a new metamaterial absorber with a structured impedance-matched composite that can overcome the challenges in underwater absorption. A structured tungsten-polyurethane composite with an impedance matched to water was used. The mass density of the dispersed tungsten particles is larger than that of water; in the meanwhile, this composite material exhibits a remarkably slower longitudinal wave speed when compared with that in water. Therefore, the Fabry-Pérot resonances with the backing layer have been achieved based on a remarkably smaller thickness than that of conventional materials. The surface impedance of each Fabry-Pérot resonator can be described as ${Z_n} = i{Z_w}\cot (\omega {L_n}/{v_c})$. Using the Mittag-Leffler expansion, the Green function is deemed the surface response of the *n_t_h* composite rod. With mathematical manipulation, *G_n_* can be denoted in the Lorentzian form


(25) \begin{eqnarray*} {G_n}(\omega ) = \frac{{{\chi _n}}}{{\Omega _n^2 - {\omega ^2} - i\beta \omega }}, \end{eqnarray*}

where χ_*n*_ = 2/(ρ_*c*_*L_n_*) = 2/(ρ_*w*_*L*_0_), the resonant frequency Ω_*n*_ = 2π*f_n_* = π*v_c_*/(2*L_n_*) and the factor β can be presented to model the dissipation. Here, the sound absorption coefficient is provided by the function *f_n_* = *f*_1_*e*^2(*n* − 1)/9^, with *f*_1_ representing the first-order Fabry-Pérot resonances at a low-cutoff frequency. The discretized length of each Fabry-Pérot resonant unit cell was *L_n_* = *v_c_*/(4*f_n_*). The overall surface impedance response of the integrated resonators positioned in parallel can be described as


(26) \begin{eqnarray*} {Z_s}(\omega ) = \frac{9}{{ - i\omega \sum _{n = 1}^9 {{G_n}} }}. \end{eqnarray*}

The sound absorption coefficient can be obtained by (24). The integrated Fabry-Pérot resonators with nine different lengths are schematically illustrated in the upper part of Fig. 9(d). Relative to the experimental data, the stainless-steel base in the simulations was considered to permit the transmission of the incident wave, which is denoted by a green dashed line in Fig. 9(d).

## CONCLUSIONS AND OUTLOOK

In this review, we have focused on reviewing the developments in the field of underwater acoustic metamaterials over the past two decades. Although acoustic metamaterials have been extensively investigated in the air, they still face significant challenges in underwater environments. The field of underwater acoustic metamaterials has expanded significantly with the development of fundamental physics and material science. We determined methods of designing metamaterial structures that can manipulate acoustic waves to meet our expectations. However, much remains to be done in underwater environments, as mentioned at the beginning of this review. The impedance ratio between the metamaterial structure and background medium in water or human tissue is approximately 3600 times that in air. This large contrast indicates that most of the energy generated by transducers or loudspeakers in water or tissue interacts with the metamaterial structure; therefore, in most cases, underwater metamaterials cannot be considered as absolutely rigid boundaries in underwater environments. Considering the solid-fluid interactions, the effective parameters are dominated by multiple scattering [4,5,36]. Complicated fluid-like metamaterials also exhibit unavoidable viscothermal effects and are therefore more challenging to design.

From the perspective of fundamental science and intelligent sensing technology, transformation theory has been proven to be an effective way to guide complicated metamaterial design [42,43]; the quasi-isotropic method based on conformal transformation reduces the anisotropic parameters to avoid complicated structure manufacturing [63]. Pentamode metamaterials in water can be regarded as metafluids that can reduce the shear moduli of microstructures. One challenge is how to avoid the scattering effects of underwater obstacles with arbitrary shapes and frequency bands to break the detection barriers and guarantee detection capabilities in oceans and rivers. Another challenge is the design of compact and miniaturized sound sources for directional acoustic detection in water and the provision of key technical support for low-power and intelligent detection systems. An underwater active phase array can achieve greater performance; however, the complex circuit systems and signal processing modules make it difficult to design a compact and integrated device.

Underwater metasurfaces, as a new field of phase engineering, show their potential for subwavelength control [48,49]. However, existing metamaterials with effective acoustic parameters can hardly effectively extend the acoustic propagation path over a large scale. Some solutions have also been proposed to solve this problem, such as membrane metamaterials and resonant-based metamaterials; however, the narrow band limitation is difficult to overcome. Additionally, there are the potentially pernicious effects that viscothermal dissipation might produce on underwater acoustic metamaterials, which are usually made of a periodic repetition of building units. The existence of viscothermal losses in the periodic structures defining the underwater acoustic metamaterial should be taken into account to properly obtain the desired behavior. Recently proposed underwater metamaterials with impedance-matched metagels and composite absorbers show their potential possibility in underwater broadband transmission and absorption [66,118]. What is the low-frequency limit of an underwater metasurface? We envision that with micro-/nanofabrication, noise suppression by underwater metasurfaces can be extended to lower-frequency bands.

Moreover, soft metamaterials can achieve remarkable and even extreme acoustic properties that benefit from material fabrication, acoustic parameter characteristics and 3D printing. Micro-/nanoscale manufacturing has been used to design extremely complex structures with relatively stable acoustic properties and can reach a negative index [128,168,169]. This new concept integrates the advantages of micro-/nanofabrication and metamaterial design. Additionally, compact soft metamaterials can produce less rigid or elastic scattering with water or human tissues. This new concept has been rapidly developed in underwater acoustics, such as metaskin insulators [170], broadband tunable metagels [66,171] and bioinspired hydrogel robots [172]. Can these soft metamaterial concepts be used to achieve underwater acoustic digital encoding for long-range detection?

The frequency and speed of acoustic waves are lower than electromagnetic waves and microwaves, and the bandwidth of underwater communication is usually limited. The proposed orbital angular momentum of acoustic vortex beams provides a new independent degree of freedom for improving the transmission rate of acoustic communication [93]. However, phase control and long-distance underwater acoustic communication in practical water environments are yet to be achieved. Existing metamaterial designs for underwater phase control are almost narrow bands and suffer diffraction limits. In other words, it is difficult for most underwater metamaterials to simultaneously achieve broadband and long-distance non-diffraction transmission. Acoustic holography is a technique for manipulating acoustic phase using a diffraction integral, which provides a new method for underwater 3D acoustic field imaging and wavefront shaping such as beam steering and collimation. What is the resolution limit of traditional phased array sources? Acoustic holography has been shown to achieve reconstruction degrees of freedom two orders of magnitude higher than commercial phased array sources [51]. How can intelligent sensing and imaging of an underwater environment be achieved based on acoustic holography?

The development of advanced exploration technology is not only related to ocean exploration but also vital to the frontier of science and technology. The oceans cover nearly three-quarters of the Earth’s surface, yet $95\%$ of the ocean has not been explored. Compared with light and electromagnetic waves, sound waves can travel long distances underwater, and low-frequency sound can even achieve thousands of kilometers of transmission. Because of the importance of the “transparent ocean” [173] and “smart ocean” proposed by the Intergovernmental Oceanographic Commission of UNESCO, there is an urgent need to overview current advances in underwater acoustic metamaterials. We envision that underwater acoustic metamaterials can provide new design schemes for developing next-general ocean technologies such as ocean acoustic tomography, ocean observing network, ocean exploration, etc.

Overall, we conclude that the future of underwater metamaterials can be applied in a water environment and biological tissues. Can acoustic metamaterials be useful in underwater acoustics or medical ultrasound? These questions require further study, but we believe that answers can be found.
